# Changes in Mitral Annular Ascent with Worsening Echocardiographic Parameters of Left Ventricular Diastolic Function

**DOI:** 10.1155/2016/6303815

**Published:** 2016-03-08

**Authors:** Paula M. Hernández Burgos, Angel López-Candales

**Affiliations:** ^1^University of Puerto Rico School of Medicine, P.O. Box 365067, San Juan, PR 00936-5067, USA; ^2^Cardiovascular Medicine Division, University of Puerto Rico School of Medicine, Medical Sciences Campus, P.O. Box 365067, San Juan, PR 00936-5067, USA

## Abstract

*Background*. While the mitral annular plane systolic excursion (MAPSE) has been suggested as a surrogate measurement of left ventricular ejection fraction, less is known about the relative value of mitral annular ascent (MAa).* Methods*. Our database was queried for complete transthoracic echocardiograms performed for any clinical indication. Baseline echocardiographic measurements were compared to determine any correlation between MAa and traditional Echo-Doppler echocardiographic measures to characterize left ventricular diastolic dysfunction (LVDD).* Results*. Patients with normal LV diastolic function were younger (41 ± 13 years) than patients with LVDD (stage 1: 61 ± 13 years; stage 2: 57 ± 14 years; and stage 3: 66 ± 17 years; *p* = 0.156). LV ejection fraction decreased in patients with stage 2 LVDD (63 ± 17%) and was further reduced in patients with stage 3 LVDD (28 ± 21; *p* = 0.003).* Discussion*. While a vigorous MAa excursion was seen in patients with stage 1 LVDD, MAa significantly decreased in stage 2 and stage 3 LVDD patients. Our results highlight the importance of atrioventricular coupling, as MAa motion seems to reflect changes in left atrial pressure. Additional studies are now required to better examine atrioventricular interactions and electromechanical coupling that might improve our assessment of LV diastolic function.

## 1. Introduction

Measuring the maximal descent of the mitral annulus during systole, commonly referred to as mitral annular plane systolic excursion (MAPSE), has been suggested as a surrogate measurement of left ventricular ejection fraction (LVEF) [1–4]. In contrast, less is known about the relative value of mitral annular ascent (MAa) that occurs late in LV diastole, particularly after atrial contraction while in normal sinus rhythm.

Given the temporal relation of the mechanical events in late diastole, we sought to determine if there was any correlation between MAa and traditional Echo-Doppler echocardiographic measures to characterize left ventricular diastolic dysfunction (LVDD) currently used as recommended by both the American Society of Echocardiography (ASE) and European Association of Echocardiography [5–7].

## 2. Methods

For this retrospective study, our echocardiographic database was queried for complete transthoracic echocardiograms performed for any clinical indication at the University of Cincinnati College of Medicine Main University Hospital Echocardiography Laboratory. Inclusion variables include normal sinus rhythm at the time of the examination, good visualization of both the LA and LV endocardium, and complete spectral Doppler study with M-mode tracings and tissue Doppler interrogation of the lateral portion of the MA with clear signals in all studies [5]. In contrast, data from patients with frequent premature or atrial contraction beats, previous myocardial infarction, valve disease worse than mild, pulmonary hypertension, previous cardiac surgery, left bundle branch block, pericardial disease, or the presence of a pacer or defibrillator wire were not included. LV diastolic function was classified as normal (0), impaired relaxation (1), pseudonormal (2), and restrictive pattern (3) following published recommendations as suggested by Nagueh and associates [5].

**Figure 1:**
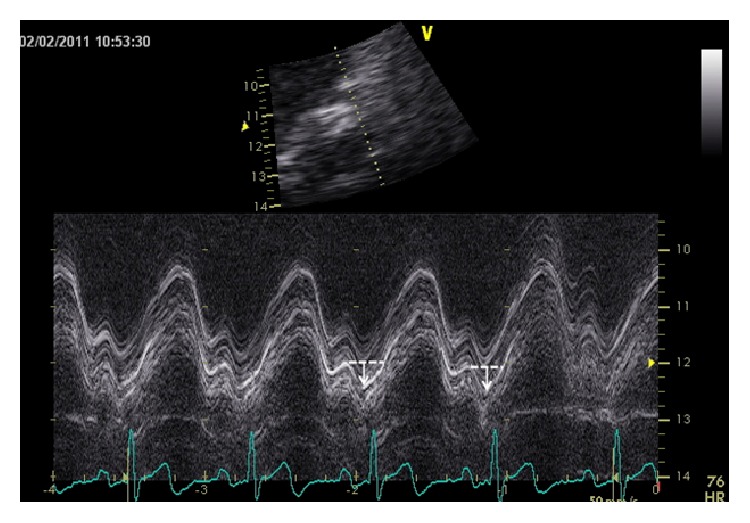
Representative M-mode tracing showing maximal mitral annular plane systolic motion. MA ascent (MAa) was measured as the distance of the annulus traversed from the end of diastasis until the end of atrial contraction (white arrow) from the imaginary line drawn (dotted white line) that represents annular motion during diastasis with the sharp ascent (downward slope) towards the atria after atrial contraction. Please note that typically several lines are always seen when performing M-mode of the MA due to the contribution of chordal structures. However, since these images have the same frequency, their reflected images maintain the same distance. Usually, the most reflective echo-bright image should be used for the purpose of measurements as shown in this figure. MAPSE is also shown.

Two-dimensional echocardiographic studies were performed using commercially available systems (Vivid 7 and Vivid 9; GE Medical Systems, Milwaukee, WI, USA). Based on previous findings by our laboratory, pulsed-wave tissue Doppler imaging (TDI) was only performed on the lateral portion of the MA for assessing maximal MA excursion and MAa [3, 4]. MAa was measured as the distance of the lateral annulus traversed from the end of diastasis until the end of atrial contraction (Figure 1) [4]. Echocardiographic parameters were calculated using the commercially available software Merge Cardio Workstation and determined by a single observer. All continuous data are presented as mean ± standard deviation and categorical data as frequencies. Baseline characteristics were compared between groups using analysis of variance (ANOVA) and Fisher's exact test for continuous and categorical data, respectively. Echocardiographic measurements were compared using two-tailed unpaired *t*-test assuming unequal variances. In order to assess reproducibility of MAa measurements, the intraclass correlation coefficient was used.

Study approval was obtained from the University of Cincinnati IRB office (Protocol number 12061302) and since this was a retrospective study, there was no need for written consent.

## 3. Results

The main echocardiographic data from 150 patients performed for any clinical indication was collected and included for final analysis as shown in Table 1. Patients with normal LV diastolic function were younger (41 ± 13 years) than patients with LVDD (stage 1: 61 ± 13; stage 2: 57 ± 14; and stage 3: 66 ± 17 years; *p* = 0.156).

Evaluation of LV systolic function demonstrated that LV ejection fraction began decreasing in patients with stage 2 LVDD (63 ± 17%) and was further reduced in those patients with stage 3 LVDD (28 ± 21; *p* = 0.003).

**Figure 2:**
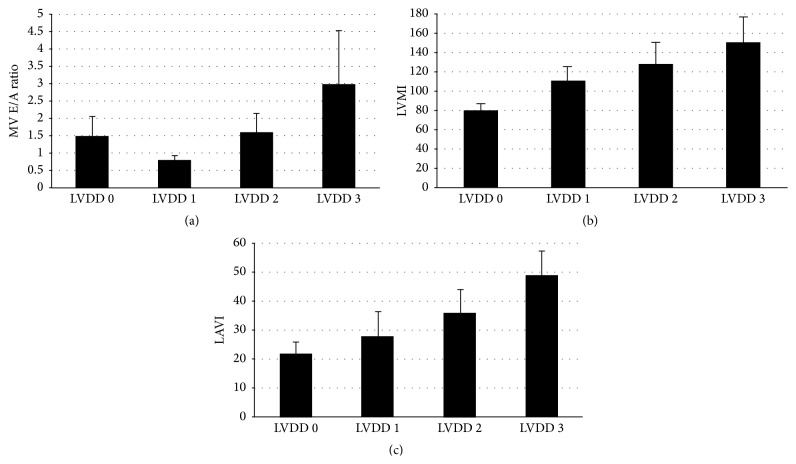
(a) Box plot showing MV E/A ratio mean and standard deviation values for each stage of LV diastolic function. (b) Box plot showing LV mass index (LVMI) mean and standard deviation values for each stage of LV diastolic function. (c) Box plot showing left atrial volume index (LAVI) mean and standard deviation values for each stage of LV diastolic function.

When evaluating other echocardiographic parameters, we found the traditional MV E/A ratio pattern with worsening LVDD (Figure 2(a)), as well as an increase in both LV mass index (Figure 2(b)) and left atrial volume index (Figure 2(c)) with worsening LVDD parameters.

Furthermore, mean and standard deviation values for MAa, lateral MA TDI A′, and MV/MA TDI E′ values for the study population according to LV diastolic function are shown in Table 2.

**Figure 3:**
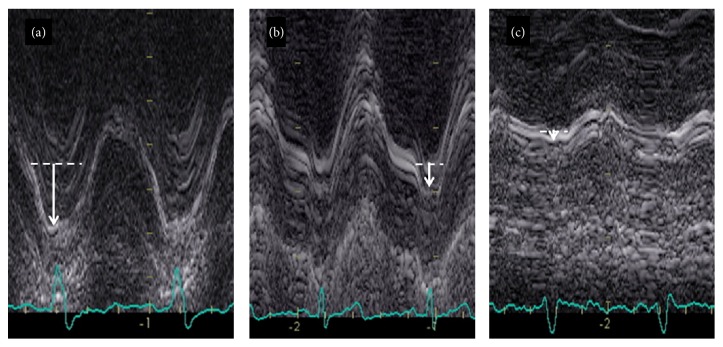
MAa M-mode images are shown to demonstrate the observed differences in MAa. (a) shows the M-mode tracing that is a representative MAa image of a patient with stage 1 LVDD. (b) demonstrates a representative MAa of a patient with stage 2 LVDD and (c) an MAa tracing of a patient with stage 3 LVDD. Solid lines represent site of measurement while the arrows represent actual MAa measure. Please note the relative magnitude of each MAa by the size of the arrow.

A representative image showing the changes in MAa excursion with regard to LV diastolic function is shown in Figure 3.

Finally, interrater agreement (*κ*, Kappa) value assessment was used to measure variability in terms of MAa measurements. The strength of the agreement was moderate (*κ* = 0.546, standard error = 0.158, and 95% CI = 0.237 to 0.855) when two untrained observers reproduced MAa measurements and was very good (*κ* = 0.985, standard error = 0.094, and 95% CI = 0.711 to 1.000) if a trained observer was performing these measurements.

## 4. Discussion

For the first time, to our knowledge, an apparent relationship between MAa motion and LVDD was identified. Specifically, a vigorous MAa excursion was exclusively seen in patients with stage 1 LVDD when compared to the rest of the studied population, including patients with normal LV diastolic function. Moreover, MAa significantly decreased in stage 2 LVDD patients, while the lowest MAa values were seen in stage 3 LVDD patients.

LV relaxation is known to occur with a concomitant active movement of the MA away from the apex [5]. The velocity of this MA motion has been demonstrated to correlate well with how fast the LV fills and relaxes [6–10]; but the effect of LV diastole with regard to the late MAa component had never been examined. From a mechanistic point of view, the first question would be why to consider this late MAa ascent motion of the annulus with regard to LV diastole. In principle, this portion of MA motion should correspond to LV compliance. Hence, problems with increased left atrial pressure would be mainly seen during this portion of diastole. Consequently, by examining this relationship we would be exploring novel information not previously investigated and also developing a new venue for examining the axiomechanical relationship known to exist between left atria and ventricle (atrioventricular interactions) all throughout the cardiac cycle.

The following are the most important limitations that need to be addressed: retrospective nature of the study, small number of patients included into the final analysis, exclusion of other pathological events that could affect MAa measurements, and lack of speckle-tracking imaging. In addition, we did not include data of MAa measurements during exercise, particularly when there are a significant number of patients that are diagnosed with LVDD during exercise [11]. However, the main goal was attained. Finally, only the lateral portion of the MA was used to measure TDI E′ velocity in order to assess global LV diastolic function. Since this was the first attempt in correlating MAa excursion with diastolic MA velocities, it would make sense to only use the lateral portion MA which is known to move the fastest and has the greatest overall displacement [7, 8].

Even though we are not proposing the use of MAa as additional measure to asses LVDD, our results highlight the importance of atrioventricular coupling as MAa motion seems to reflect changes in LA pressure, known to occur with worsening LVDD. This perhaps suggests the need for a more comprehensive integration of atrioventricular interactions when assessing LV diastolic function.

## Figures and Tables

**Table 1:** Clinical indication to perform echocardiogram.

Main indications	LVDDD 0	LVDD 1	LVDD 2	LVDD 3
Hypertension	2	23	22	0
Postcerebrovascular accident	4	8	0	0
Pulmonary causes	5	5	0	0
Renal causes	3	9	7	2
Coronary disease	0	5	3	0
Heart failure	0	0	8	0
Other	21	5	0	18

**Table 2:** Results for MAa, lateral MA TDI A′, and MV/MA TDI E′ values for the study population according to LV diastolic function.

Variables	LVDD 0 (*N* = 35)	LVDD 1 (*N* = 55)	LVDD 2 (*N* = 40)	LVDD 3 (*N* = 20)	ANOVA
Lateral MA TDI A′	8 ± 2	11 ± 3	7 ± 3	4 ± 2	0.01
MV E/MA TDI E′ ratio	5.6 ± 2.6	9.4 ± 4.8	15.1 ± 7.2	21.0 ± 14.6	<0.001
MAa	0.46 ± 0.13	0.61 ± 0.14	0.41 ± 0.13	0.18 ± 0.5	<0.001
